# HIV Infection and Exposure Increases Cariogenic Taxa, Reduces Taxonomic Turnover, and Homogenizes Spatial Differentiation for the Supragingival Microbiome

**DOI:** 10.21203/rs.3.rs-4720457/v1

**Published:** 2024-08-10

**Authors:** Allison E. Mann, Ciara Aumend, Suzanne Crull, Lauren M. O’Connell, Esosa Osagie, Paul Akhigbe, Ozoemene Obuekwe, Augustine Omoigberale, Matthew Rowe, Thomas Blouin, Ashlyn Soule, Colton Kelly, Robert A. Burne, Modupe O. Coker, Vincent P. Richards

**Affiliations:** University of Wyoming; Clemson University; Clemson University; Clemson University; Institute of Human Virology; Institute of Human Virology; University of Benin Teaching Hospital; University of Benin Teaching Hospital; Clemson University; Clemson University; Clemson University; Clemson University; University of Florida; Rutgers University; Clemson University

**Keywords:** oral microbiome, HIV, longitudinal analysis, caries disease, CD4 count

## Abstract

**Background:**

The oral microbiome comprises distinct microbial communities that colonize diverse ecological niches across the oral cavity, the composition of which are influenced by nutrient and substrate availability, host genetics, diet, behavior, age, and other diverse host and environmental factors. Unlike other densely populated human-associated microbial ecosystems (e.g., gut, urogenital), the oral microbiome is regularly and directly exposed to the external environment and is therefore likely less stable over time. Cross sectional studies of the oral microbiome capture a glimpse of this temporal dynamism, yet a full appreciation of the relative stability, robusticity, and spatial structure of the oral environment is necessary to understand the role of microbial communities in promoting health or disease.

**Results:**

Here we investigate the spatial and temporal stability of the oral microbiome over three sampling time points in the context of HIV infection and exposure. Individual teeth were sampled from a cohort of 565 Nigerian children with varying levels of tooth decay severity (i.e., caries disease). We collected 1,960 supragingival plaque samples and characterized the oral microbiome using a metataxonomic approach targeting an approximately 478 bp region of the bacterial *rpo*C gene. We found that both infection and exposure to HIV have significant effects on the stability of the supragingival plaque microbiome at both the spatial and temporal scale. Specifically, we detect (1) significantly lower taxonomic turnover of the oral community among exposed and infected children compared to unexposed children, (2) we find that HIV infection homogenizes the oral community across the anterior and posterior dentition, and (3) that impaired immunity (i.e., low CD4 count) and low taxonomic turnover over time in children living with HIV is associated with higher frequency of cariogenic taxa including *Streptococcus mutans*.

**Conclusions:**

Our results document substantial community fluctuations over time in children unexposed to HIV independent of oral health status. This suggests that the oral community, under typical conditions, rapidly adapts to environmental perturbations to maintain homeostasis and that long-term taxonomic rigidity is a signal of community dysfunction, potentially leading to a higher incidence of oral disease including caries.

## BACKGROUND

The human oral microbiome is a highly dynamic and personalized ecological system composed of heterogeneous microhabitats (e.g., saliva, cheeks, tongue, hard and soft palate, teeth, and gums) inhabited by diverse bacteria, archaea, microbial eukaryotes, and viruses. The most densely populated of these microhabitats are microbial biofilms that form on the teeth (i.e., dental plaques), which harbor a distinct community as compared to those formed on soft tissues. The supragingival plaque microbiome is further shaped by differences in substrate and oxygen availability, salivary flow, and the presence of other co-aggregating taxa [1–4]. Along with features of the oral environment that can be attributed to host genetics (e.g., morphology of the oral tissues, salivary production rate, composition, and pH, and host immune response), the composition and stability of the oral microbiome is heavily influenced by diet, behavior (e.g., oral hygiene practices, smoking), and age [5–8]. Dental plaques form on a tooth through a series of ordered colonization events, resulting in a highly structured, species rich microbial biofilm [9, 10]. Community homeostasis of the plaque microbiome is essential for the maintenance of good oral health [9]. However, dental plaques are also in direct and regular contact with an individual’s external environment and are thus more susceptible to punctuated ecological shifts over time due to changing environmental conditions [8, 11, 12]. The relative resiliency of the oral environment to these shifts has important implications for the maintenance of good oral health and the prevention of microbial dysbiosis.

Dysbiosis of the oral environment is associated with a variety of common chronic diseases, the most prevalent of which is tooth decay. If untreated, tooth decay results in the formation of carious lesions in the enamel surface, bacterial infection of the dentin, pulp cavity, and surrounding tissues, and eventually, tooth loss. Severe infection of a tooth can cause sepsis and even death. Untreated dental caries in permanent teeth is the most common chronic disease, globally affecting an estimated 2.5 billion people, despite it being a largely preventable condition [13]. The etiology of caries is initiated and exacerbated by ecological shifts in the plaque microbial community – driven by environmental changes in the oral cavity – and typically results in a reduction of overall community richness and complexity, though some studies have found an increase in diversity among teeth with active carious lesions [14–16]. While there are specific taxa that are directly implicated in the development or intensification of caries disease progression (primarily *Streptococcus mutans* and other aciduric and acidogenic taxa), it is generally accepted that caries is the result of a population imbalance in the oral microbial community [17–20], and that risk of caries disease is related to the structure, stability, and resilience of the community to ecological stressors [9, 21].

Children living with HIV have a higher prevalence and more severe clinical presentation of dental caries in both primary and permanent dentition, the impact of which is associated with viral load and individual immune status [22–27]. Previous work by our group found a small but distinct effect of HIV status on the supragingival plaque microbiome which is exacerbated in the context of severe caries [28]. Moreover, there is growing evidence that perinatal exposure to HIV has systemic effects on the health, growth, and development of children [29–35] and previous work by our group found a distinct oral mycobiome [36], lower salivary flow and decreased salivary pH [37], increased developmental defects of the enamel surface [38], and increased incidence of stunting in both children exposed to and infected by HIV [Manuscript in preparation].

Given the relative susceptibility of the oral microbiome to perturbations as compared to other microbe-rich host-associated environments, cross-sectional studies of the oral microbiome only provide a brief snapshot of the ecological diversity and dynamics of the oral microbiome over time, and the impact of illness or stress on the long-term stability of the oral microbiome is poorly defined. In the current study, using three time points, we compare the temporal and spatial stability of the oral microbiome among children living with HIV (HI), children perinatally exposed to but uninfected by the virus (HEU), and unexposed and therefore uninfected (HUU) children. We find significantly lower taxonomic turnover in HEU and HI children as compared to HUU children over time. While this pattern in HI children is predominantly due to a higher prevalence of diseased teeth with low community complexity, we detect significantly lower taxonomic turnover for HEU children even when only healthy teeth are compared.

In addition to temporal patterning of the plaque microbiome, we find that spatial patterning of the bacterial community across the dental anatomy is disrupted in children living with HIV as compared to HEU and HUU children wherein children exposed to or uninfected by HIV have a clear differentiation between the communities inhabiting the anterior and posterior teeth but there is no such separation among HI children. Finally, we document an initial increase in CD4 counts in HI children from visit one to visit two, followed by a slight decrease in visit three, possibly a result of ongoing anti-retroviral treatment and increasing age of the cohort. Importantly, increasing CD4 counts among HI children is associated with a reduction of cariogenic taxa. Our results document the importance of longitudinal and spatial sampling of the supragingival plaque microbiome for a more holistic appreciation of the role of oral bacteria in the development of caries in children living with HIV.

## METHODS

### Study design and sample collection.

Samples included in this study were collected from 565 children between May, 2019 and February, 2021 during three separate clinical visits at the University of Benin Teaching Hospital in Benin City, Nigeria. In total, we collected 1,960 supragingival plaque samples as part of the Dental Caries and its Association with Oral Microbiome and HIV in Young Children – Nigeria (DOMHaIN) Study [25]. A total of 614 supragingival plaque samples were collected from uninfected and unexposed children (HUU), 611 from children exposed to HIV in utero but uninfected with HIV (HEU), and 735 from children living with HIV (HI).

Supragingival plaque samples were collected from a single tooth and categorized into one of six progressive health categories. First, the sample was categorized according to the condition of the tooth of origin using the International Caries Detection and Assessment System [39] as either originating from (1) a tooth with no cavity (H; ICDAS score = 0), (2) a tooth with an active enamel lesion (E; ICDAS score = 1 to 3), or (3) a tooth with an active dentin carious lesion (D; ICDAS score ≥ 4). We next characterized individual plaque samples according to the observed caries experience of the child at the time of sampling as either (1) caries-free (CF), i.e., no clinical or reported evidence of caries (number of decayed, missing, and filled teeth [DMFT] = 0), (2) caries active with lesions present in the enamel of the tooth only (CE; DT = 0, MFT ≥ 0), or (3) caries active with at least two teeth presenting with active dentin lesions (CD; DT ≥ 2, MFT ≥ 0). Together, these two classification schemes were used to place each sample into one of six progressive disease states as follows: (1) plaque from a healthy tooth collected from a child with no caries (H-CF, n = 1,169), (2) plaque collected from a healthy tooth collected from a child with active enamel caries (H-CE, n = 119), (3) plaque collected from a healthy tooth collected from a child with active dentin caries (H-CD, n = 251), (4) plaque collected from a tooth with an active enamel cavity from a child with active enamel cavities (E-CE, n = 70), (5) plaque collected from a tooth with an active enamel cavity from a child with active dentin cavities (E-CD, n = 110), and (6) a plaque collected from a tooth with an active dentin cavity from a child with active dentin cavities (D-CD, n = 241). In brief, we collected each plaque using a sterile curette which was then stored in a sterile 2 mL cryogenic vial containing 500 μL of RNAlater. Post-collection, samples were placed immediately on ice and stored at −80°C within two hours of collection. Detailed sampling procedures are described in Coker et al. [25].

### DNA extraction, library preparation, and sequencing

We extracted DNA from each sample using the DNeasy PowerBiofilm kit (Qiagen, Valencia, CA, USA) following the manufacturer’s suggested protocol. We quantified the total DNA yield post-extraction for each sample using a Qubit fluorometer (Invitrogen, Carlsbad, CA). To track potential sources of contamination, an extraction blank using molecular-grade water was processed in parallel to all samples. To characterize the bacterial community, we amplified a fragment of the bacterial *rpo*C gene using custom primers (rpoCF: 5’ – MAYGARAARMGNATGYTNCARGA – 3’, rpoCR: 5’ – GMCATYTGRTCNCCRTCRAA – 3’) as described in Mann et al. [28]. Each PCR reaction consisted of the following: 0.5 μL each of the forward and reverse primers, 10 μL molecular grade water, 4 μL of template DNA, and 10 μL of the Platinum Hot Start PCR Master Mix (Invitrogen, Carlsbad, CA, USA). We processed PCR blanks (molecular grade water) in parallel to all samples to track sources of contamination. Each reaction was amplified using the following thermocycler conditions: 94°C for 3 minutes followed by 41 cycles of 94°C for 45 seconds, 39.5°C for 1 minute, and 72°C for 1 minute 30 seconds. A final elongation step was performed for 10 minutes at 72°C. Amplification of all samples was confirmed through both gel electrophoresis and Qubit fluorometer (Invitrogen, Carlsbad, CA, USA). Finally, we pooled each sample library at equimolar concentrations and sequenced the final pools on an Illumina MiSeq using V3 2×300 paired-end chemistry (Illumina, San Diego, CA, USA).

### Computational analyses

We first removed primers and adapter sequences from our raw sequencing read files using Cutadapt (v 1.18) [40]. Next, we quality filtered, merged, generated Amplicon Sequence Variants (ASVs), and removed suspected chimeric sequences using DADA2 (v 1.22.0) [41] in an R (v 4.1.0) environment [42]. Quality-filtered paired-end reads that were shorter than 450 bp after merging were removed from downstream analysis. We next assigned a taxonomy to each ASV using Kraken2 (v 2.1.2) [43] and a custom *rpo*C database as reference [28]. ASVs that could not be assigned to the phylum level or below (i.e., kingdom only) were removed from analysis. We removed low-abundance ASVs with a prevalence threshold of 0.1% (n = ~ 2 samples) unless they had a total abundance of more than 1,000 reads, in which case the ASV was retained for downstream analysis. Finally, we removed samples with fewer than 5,000 reads post-filtering. Rarefaction curves for all samples post-quality filtering can be found in Figure S1.

We next performed diversity analyses for the filtered dataset using the R libraries phyloseq (v 1.38.0) [44], vegan (v 2.6–4) [45], compositions (v 2.0–6) [46], and microbiome (v 1.16.0) [47]. Significance in diversity metrics between groups was determined using PERMANOVA analysis (vegan::adonis2) performed on Center Log-Ratio (CLR) transformed data. We used coda4microbiome (v 0.1.4) [48] to identify microbial signatures that are predictive of sample metadata. This method calculates the minimum number of features (here, bacterial species) that have the maximum predictive power for a particular user-defined metadata category. A microbial signature, therefore, is defined by the relative abundance of two groups of taxa wherein the balance of taxa with positive and negative coefficients has the highest correlation with your chosen variable.

We calculated the taxonomic turnover of the oral microbiome on a single tooth over time following the protocol detailed in Bastiaanssen et al. [49]. In brief, we defined taxonomic turnover as the absolute Euclidean distance between paired plaque samples from the same tooth and individual over two or more collection time points using an Aitchison distance matrix calculated from CLR transformed count data. Significance between groups was determined using a Wilcoxon rank signed test.

Next, we wanted to identify conserved microbial associations over time (i.e., the co-occurrence of species pairs on distinct teeth/samples) within and between our HIV status and tooth health groups. To do this, we first generated microbial association networks with NetCoMi (v 1.1.0) [50] and Spiec-Easi (v 1.1.3) [51] using the neighborhood selection method (MB) [52]. Before network generation, we collapsed our ASV frequency table to the species level and only included species found in at least 1% of all samples with a minimum of 10 observations to minimize the effect of low frequency taxa. We generated microbial association networks for each HIV status (HI, HEU, HUU) and tooth health group (H, E, D) at each sampling time point (three networks per group) and used anuran (v 1.1.0) [53] to depict a single Core Association Network (CAN) for each HIV status or tooth health group across all time points (set size = 1.0). Anuran identifies conserved patterns (i.e., groupings) across networks. As such, CANs generated for this study represent the consensus of microbial associations over time within our groups. In addition, we generated a “global” CAN using all samples to act as a baseline comparison to our group-specific CANs (set size 0.6). Next, we used the greedy clustering algorithm implemented in igraph (v 1.6.0) [54] to cluster species and quantify cluster modularity within each CAN [55]. Only clusters with at least ten or more members were analyzed.

Finally, we wanted to better understand the community ecology on individual teeth directly before or after the tooth has a high relative abundance of the cariogenic taxon *S. mutans*. While *S. mutans* is a dominant taxon in many supragingival plaque samples collected from late-stage cavitated teeth, it is often absent or at very low frequency at the early stages of tooth decay (i.e., enamel lesions or white spots) [28, 56, 57]. As such, it is unclear what role (if any) *S. mutans* or other bacterial groups play in the initiation of caries. Conversely, many late-stage cavitated teeth have very little or no *S. mutans* which may suggest that the proliferation of *S. mutans* during the intensification of tooth decay is self-limiting and the community collapses over time [28]. As such, it is important to know if there are predictable taxa that recolonize the tooth and if this recolonization recapitulates the original plaque community.

To better understand the plaque environment before or after the proliferation of *S. mutans*, we first identified all individual teeth that were sampled from the same child at more than one time point. From this, we identified teeth that had a relatively low proportion of *S. mutans* ( < = 5%) either before or after the same tooth had a relatively high proportion of *S. mutans* ( > = 10%). Using this subset of paired samples of the same tooth over time, we first performed a random forest classification analysis using ranger (v 0.16.0) [58] to determine if the microbial community can be used to predict later *S. mutans* colonization and proliferation (i.e., before *S. mutans*) or if the community that recolonizes the tooth after *S. mutans* collapse is consistent and predictable (i.e., after *S. mutans*). Finally, we used a post-hoc analysis of our random forest classification model to identify specific bacterial species that are explanatory of our two groups (either before or after *S. mutans*) using FastShap (v 0.1.1) [59].

Conda environments and all scripts for data analysis can be found at https://github.com/aemann01/long_oral_microbiome and are archived at Zenodo (DOI: 10.5281/zenodo.11396311) for analytical reproducibility.

## RESULTS

### Sample demographic and taxonomic summary.

After quality filtering and removal of samples with low read counts, we retained 14,111 unique ASVs assigned to a total of 1,960 individual supragingival plaque samples collected from 565 children. Our final sample demographic includes 900 samples collected from female participants and 1,060 from male participants. Of the plaque samples, 746 were collected at visit one, 596 at visit two, and 618 at visit three. The average age of participants at visit one was 6.9 years old (SD ± 1.9) and 7.2 years old (SD ± 2.0) at visit three. Of all plaque samples retained post-quality filtering, 38% originated from an HI participant, 31% from a HEU participant, and 31% from a HUU participant. Full sample metadata can be found in Table S1. The top phyla found across all samples and all visits included *Bacteroidetes* (average proportion 31%), followed by *Firmicutes* (26%), *Proteobacteria* (21%), *Actinobacteria* (16%), and *Fusobacteria* (15%). Top genera include *Streptococcus* (66%), *Ligilactobacillus* (64%), *Rothia* (56%), *Capnocytophaga* (55%), and *Prevotella* (55%) (Table S2). HEU samples across all three visits had significantly higher alpha diversity as measured by the observed number of ASVs as compared to HI samples (p = 0.017) but not compared to HUU samples, and D-CD samples had significantly lower alpha diversity as measured by Shannon diversity as compared to E-CD and any healthy tooth independent of the overall oral health (p < 0.0001) (Figure S2).

### Children unexposed and uninfected with HIV have a higher rate of taxonomic turnover over time as compared to other children in this cohort.

**Figure 1:**
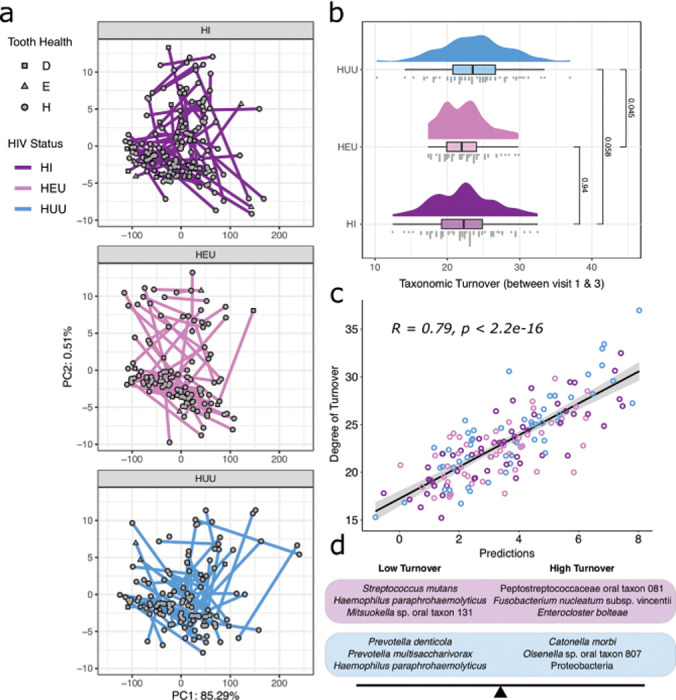
Taxonomic turnover on individual teeth is higher in HUU as compared to HEU and HI children. (a) Principal coordinate plot of individual teeth collected from visit one and visit three for each of the three HIV status groups. Lines connect plaque samples collected from the same tooth and same individual. Point shape indicates tooth health at the time of sampling. (b) Distribution of volatility scores for each of the three groups at visit one versus visit three. (c) Predictions plot of the microbial signature model generated using all teeth with more than one sampling point (visit one, two, and three). Correlation coefficient and p value calculated from all samples in the plot independent of HIV status. (d) The top three weighted taxa in the microbial signature model for high or low volatility for HI children (purple) and HUU children (blue). There were no significant taxa detected for HEU children.

We defined taxonomic turnover on the same tooth over time as the absolute Euclidean distance between paired plaque samples using an Aitchison distance matrix. Teeth with high taxonomic turnover will be less similar over time while those with low taxonomic turnover will be more similar. A total of 184 teeth were sampled at both visit one and visit two with an average of 182 days between sampling periods, 255 individual teeth were sampled at both visit two and visit three with an average of 222 days separating sampling visits, and 203 individual teeth were sampled at both visit one and visit three with an average of 403 days between sampling visits. Among all teeth from all individuals, we detected a high degree of taxonomic turnover with no significant differences between the HIV status groups comparing visit one and visit two or visit two versus visit three. We did, however, detect a significant difference in taxonomic turnover between groups when comparing visit one and visit three. When comparing all teeth independent of individual tooth health status, we detected a significantly higher degree of taxonomic turnover among teeth sampled from HUU children as compared to teeth sampled from HEU children (p = 0.045) and a moderately higher degree of taxonomic turnover when compared to HI children (p = 0.058) (Fig. 1a, 1b). Comparing only healthy teeth (H), however, we detected a significant increase in turnover among teeth collected from HUU children as compared to HEU children (p = 0.049) but no significant difference comparing HUU and HI children (p = 0.154) or HI to HEU children (p = 0.67).

We next identified microbial signatures of high or low taxonomic turnover across any tooth with two or more sampling time points. The resulting microbial signature is defined by the relative abundance of two groups of taxa where taxa with negative coefficients are correlated with low taxonomic turnover and those with positive coefficients are correlated with high taxonomic turnover. The absolute value of the coefficient is reflective of the degree of impact of that taxon on the model. From this analysis, we found a positive linear association between the degree of taxonomic turnover and the resulting microbial signature prediction (R = 0.79, p < 2.2e-16) (Fig. 1c). Among samples from children living with HIV, taxa associated with low taxonomic turnover include *S. mutans* (coeff: −0.42), *Haemophilus paraphrohaemolyticus* (coeff: −0.21), *Mitsuokella* sp. oral taxon 131 (coeff: −0.15), *Prevotella multisaccharivorax* (coeff: −0.14), and *Neisseria cinerea* (coeff: −0.08) (Fig. 1d). Taxa associated with high taxonomic turnover in children living with HIV include Peptostreptococcaceae bacterium oral taxon 081 (coeff: 0.29) followed by *Fusobacterium nucleatum* subsp. vincentii (coeff: 0.24), *Enterocloster bolteae* (coeff: 0.21), *Prevotella intermedia* (coeff: 0.16), *Leptotrichia buccalis* (coeff: 0.08), and *Treponema phagedenis* (coeff: 0.02) (R = 0.8, p = 3.9e-13). Among HUU children, *Prevotella denticola* (coeff: −0.5), *P. multisaccharivorax* (coeff: −0.43), and *H. paraphrohaemolyticus* (coeff: −0.07) are associated with low taxonomic turnover while *Catonella morbi* (coeff: 0.6), *Olsenella* sp. oral taxon 807 (coeff: 0.15), an unknown species of Proteobacteria (coeff: 0.15), and *Solobacterium moorei* (coeff: 0.09) are associated with high taxonomic turnover (Fig. 1d; Table S4). We detected no microbial signature of taxonomic turnover among HEU children.

### Despite differences in taxonomic turnover on individual teeth, across all samples there are groups of species that are consistently co-associated over time.

**Figure 2:**
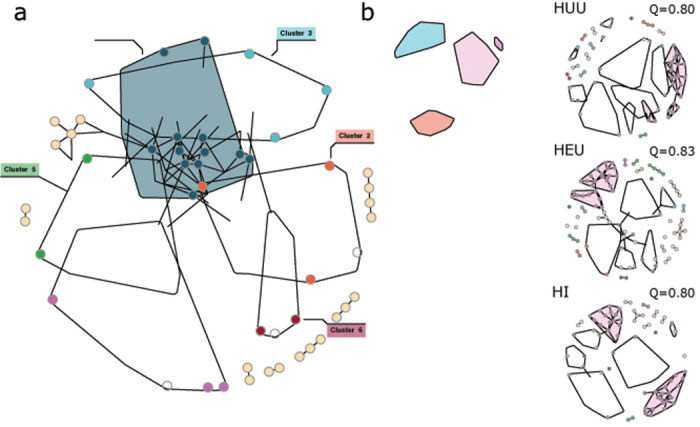
High modularity of core association networks (CAN) associated with early and late-stage caries disease. (a) Global CAN generated by comparing all samples across all three visits. We identified six clusters of co-associated taxa with variable predicted functional outcomes. Cluster six includes taxa commonly associated with caries disease (red). Clusters defined by this global CAN network are used to color code HIV status group and individual tooth health networks (b & c). (b). CAN networks by individual tooth health. Healthy teeth (H) have a substantially more interconnected network with low modularity (Q). Both teeth with enamel lesions (E) and dentin cavities (D) have extremely sparse and poorly connected networks with high modularity. (c) CAN networks across all three visits grouped by HIV status. Modularity of each network denoted for each CAN network (Q). Polygons grouping clusters on each network are specific to that HIV status group CAN network. Colors represent cluster identity from global CAN (a). White nodes are nodes unique to that CAN.

We next used core microbial association networks (CANs) to identify clusters of species that are consistently co-associated with one another on teeth over all three clinical visits. First, we created a “global” CAN generated from all plaque samples across all three visits to act as a baseline comparison to group-specific CANs. In our global CAN we detected six distinct cluster communities, the largest of which were Cluster 2 (n = 35), Cluster 3 (n = 38), and Cluster 4 (n = 39) and the smallest, Cluster 6 (n = 10) (Fig. 2a; Table S5). In general, co-associated species within clusters appear to have similar functional or clinical relevance. For example, commensal and structural plaque species (i.e., bacteria previously described as important in biofilm formation and structure) dominate Cluster 4 (e.g., *Streptococcus sanguinis, Streptococcus gordonii, Neisseria mucosa, Haemophilus parainfluenzae, Streptococcus oralis, Streptococcus mitis*, and *Corynebacterium durum*) and Cluster 5 (e.g., *Leptotrichia* sp. oral taxon 215, *Leptotrichia* sp. oral taxon 212, *Corynebacterium matruchotii, Streptococcus cristatus*) [60–62] while Cluster 1, Cluster 2, and Cluster 3 include a mixture of suspected commensal and potential pathogenic species. For example, Cluster 2 includes a variety of periodontal pathogens including members of the classic “red complex” in the etiology of periodontal disease (i.e., *Treponema denticola, Tannerella forsythia*, and *Porphyromonas gingivalis*) as well as species that previously have been isolated from periodontal pockets or coaggregate with other periodontal pathogens including *Eubacterium nodatum, Eubacterium saphenum, Filifactor alocis, Porphyromonas endodontalis*, and *Treponema medium* [63–68]. The smallest cluster, Cluster 6, includes species almost exclusively associated with caries disease including *S. mutans, Scardovia wiggsiae, Propionibacterium acidifaciens, P. multisaccharivorax, P. denticola*, and *Scardovia inopinata* [15, 57, 69–75].

Next, to better understand how these core association networks differ across tooth health and HIV status groups, we calculated community modularity (Q) across all three visits within individual tooth health and HIV status groups. Modularity is a quantitative measure of network community structure wherein networks with high community modularity have more distinct (but potentially smaller) clusters that are themselves densely connected to other members of that cluster and at the same time are only loosely connected (or disconnected) from other clusters [76]. Conversely, low community modularity is reflective of fewer distinct, but potentially larger clusters of densely connected taxa. As our networks represent a consensus of co-associated taxa across all three sample visits, we expect that low community modularity (i.e., fewer distinct cluster groups) reflects higher core taxonomic stability over time.

We find that community modularity among all healthy teeth (H) is relatively low (Q = 0.75) and increases as the disease progresses to enamel lesions (Q = 0.87) and eventually to dentin lesions (Q = 0.95) (Fig. 2b). Within HIV groups, modularity of both our HUU and HI CAN networks were equivalent at Q = 0.80 while our HEU CAN network had slightly higher modularity at Q = 0.83 (Fig. 2c). This suggests that while the bacterial community inhabiting individual teeth among HEU children changes little over time (i.e., low turnover), the community is less cohesive and more fragmented. Moreover, Cluster 6 is completely absent from the HEU CAN network and conversely is the only of the three HIV status groups to have a substantial cluster representative of global Cluster 2, potentially indicative of differences in susceptibility to caries vs periodontal disease.

### High S. mutans on an individual tooth is preceded by taxa typically associated with health and does not recapitulate the original community after S. mutans community collapse.

**Figure 3:**
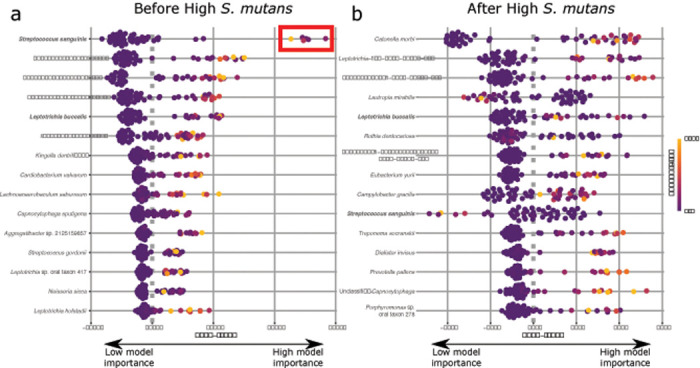
Taxa indicative of the community composition before and after high *S. mutans* relative abundance on a single tooth. Bee plots illustrate the importance of specific taxa in our random forest classification model to identify either (a) teeth before the proliferation of *S.* mutans or (b) teeth after the proliferation of *S. mutans*. SHAP value (SHapley Additive exPlanations) indicates the degree of importance for each variable on the model. Positive SHAP values indicate that that bacterial species is important for identifying the sample as belonging to the group in question while negative SHAP values indicate that the species is more important for the opposite group (i.e., before or after *S. mutans*). A SHAP value of zero indicates no impact on the model. For example, the red box in subpanel a is highlighting a subset of samples for which *Streptococcus sanguinis* is highly predictive of the sample being before the proliferation of *S. mutans*. Only the top ten taxa for either before or after high *S. mutans* relative abundance are shown. Color of points indicate how the feature value for that individual compared to the average for the entire population in which high feature value is indicated by lighter colored points. Bolded species names indicate species found in both Shapley plots.

Evidence from cross-sectional studies (e.g.,[28, 57] suggest that caries disease progression is characterized by a rapid propagation of *S. mutans* and other acidogenic/aciduric bacteria during late-stage tooth decay, followed by a collapse of the community, and eventual recolonization. For our next analysis, we wanted to determine if this process is preceded or followed by predictable taxa or groups of taxa in the plaque community. To better understand the temporal dynamics of the oral microbiome before and after high levels of *S. mutans*, we performed a random forest classification and post-hoc explanatory analysis on individual teeth with low *S. mutans* ( < = 5%) either before or after the community on the same tooth had a high level of *S. mutans* ( > = 10%). Our random forest model had high classification accuracy for teeth with high *S. mutans* (during high *S. mutans*: 83% correct) but had relatively low predictive accuracy for teeth designated as “before” or “after” high *S. mutans*. Accurate classification of teeth after high *S. mutans* was only 47% with most being misclassified as “during” and none as “before”. Teeth before high *S. mutans* were only classified correctly in 33% of cases with most being misidentified as during (50%) or after (17%). Taxa that were associated with teeth before high *S. mutans* include a variety of commensal species including *S. sanguinis, S. cristatus, S. gordonii, Abiotrophia defectiva, Aggregatibacter aphrophilus*, and *L. buccalis* as well as suspected opportunistic pathogens (e.g., *Leptotrichia shahii, Cardiobacterium valvarum, Kingella dentrificans*) (Fig. 3). Interestingly, the community after high *S. mutans* is distinct from that found before high *S. mutans* with the top explanatory taxa including *Cantonella morbi*, *Leptotrichia* sp. oral taxon 215, and *Bacteroidetes* oral taxon 274 (Fig. 3). Importantly, the lack of *S. sanguinis* is indicative of the community after colonization of high abundance of *S. mutans* which suggests that the community does not recover to its previous state, at least not initially or within the period sampled here. More fine-grained longitudinal sampling is necessary to elucidate some of these patterns over time.

### HIV infection homogenizes the plaque microbiome across the posterior and anterior dentition.

**Figure 4:**
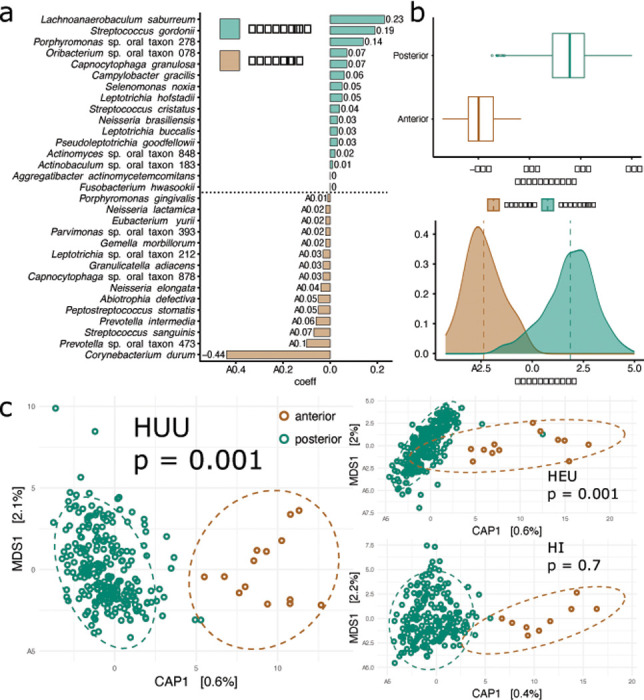
Community stratification of the anterior and posterior dentition is clear among HUU and HEU children but is dismantled in HI children. (a) Microbial signature of the anterior and posterior teeth among all individuals independent of HIV status. The relative weight of each taxon is listed as its coefficient value (y axis). (b) Prediction box and density plots of the microbial signature model for anterior and posterior teeth driven by the balance of taxa listed in subplot a. (c) Capscale plot depicting the Aitchison distance of anterior and posterior teeth in HUU, HEU, and HI children. Significance between groups determined by PERMANOVA analysis and listed as Bonferroni adjusted p values. Only adult H-CF teeth were included in this analysis.

Next, we investigated the impact of HIV status on the spatial distribution of the microbial community across the dentition of adult teeth with no carious lesions (H-CF) from all three visits. We focused on healthy teeth only for this analysis to eliminate the effect of differences in oral health among the children. We detected conspicuous differentiation among the bacterial community colonizing the anterior dentition (i.e., central and lateral incisors, canines) as compared to the posterior dentition (i.e., premolars and molars) across all adult teeth with the posterior teeth exhibiting a higher relative abundance of *Lachnoanaerobaculum saburreum, S. gordonii, and Porphyromonas* sp. oral taxon 278 and a more minor contribution of species belonging to the genera *Capnocytophaga, Campylobacter, Selenomonas, Leptotrichia, Streptococcus, Neisseria, Pseudoleptotrichia, Actinomyces, Actinobaculum, Aggregatibacter*, and *Fusobacterium*. Anterior teeth, conversely, were strongly associated with *C. durum* followed by *Prevotella* sp. oral taxon 473, and *S. sanguinis* followed by species belonging to the genera *Prevotella, Peptostreptococcus, Abiotrophia, Neisseria, Capnocytophaga, Granulicatella, Leptotrichia, Gemella, Parvimonas, Eubacterium*, and *Porphyromonas* (Fig. 4a, 4b). Importantly, however, this differentiation is primarily driven by HEU and HUU samples where there is a clear distinction between the anterior and posterior community composition (pairwise PERMANOVA with Bonferroni adjusted p value, HUU: R^2^ = 0.6, p = 0.001; HEU: R^2^ = 0.06, p = 0.001). Conversely, while the oral community living on anterior and posterior teeth among children living with HIV show the same Capscale clustering pattern, the difference between communities is not significant (pairwise PERMANOVA with Bonferroni adjusted p value, R^2^ = 0.4, p = 0.7) (Fig. 3c). Additionally, differences between the anterior and posterior oral microbiome in HUU and HEU children are predicted by fewer taxa than in HI children. Among HUU children, posterior teeth are associated with a higher relative abundance of *Neisseria weaveri, L. saburreum, Actinomyces* sp. oral taxon 848, and *Capnocytophaga granulosa* using coda4microbiome balance analysis. In HEU children, posterior teeth are strongly associated with *L. saburreum* only. Anterior teeth in both HEU and HUU children are strongly associated with *C. durum*. In HI children, 25 taxa are needed to differentiate between the anterior and posterior teeth and while *C. durum* also is the highest predictive taxon for anterior teeth in HI children, *L. saburreum* is not associated with posterior teeth prediction.

### Depressed immune status is associated with a higher prevalence of cariogenic taxa.

We next investigated the correlation of CD4 counts on the oral microbiome across all three visits. Across all samples, CD4 counts among HI children are significantly lower as compared to both HEU (p < 0.0001) and HUU children (p < 0.0001). Considering plaque samples collected at each visit, however, CD4 counts among children living with HIV (HI) significantly increased between visit one and visit two (p = 0.00016) and slightly decreased again between visit two and visit three (p = 0.014) (Figure S3). We detected no significant differences in CD4 count among HEU children between all three visits. Interestingly, HUU children had a slight but significant decrease in CD4 counts between visit one and visit three (p < 0.0001) and between visits two and three (p = 0.00016), a pattern also observed among HI children between visits two and three.

Finally, we identified microbial signatures that were most predictive of CD4 counts in children living with HIV across our three sampling periods. We found that as CD4 counts increased over the three visits, the predictive power of microbial taxa decreased with visit one having the highest correlation coefficient (R = 0.6, p < 2.2e-16) followed by visit two (R = 0.53, p < 2.2e-16) and the lowest correlation coefficient at visit three (R = 0.38, p = 4.3e-09) (Figure S4). Taxa predictive of the lowest CD4 counts among children at visit one where the mean CD4 count is the lowest of our three sampling periods (775 ± 472) include a variety of taxa involved or associated with the progression of caries disease including *S. mutans, Leptotrichia wadei, and L. saburreum* [77]. Conversely, high CD4 counts are associated with a variety of taxa previously identified as potentially protective against caries development (e.g., *Leptotrichia* sp. oral taxon 212 [78]) but also taxa that have been identified with higher caries risk (e.g., Lachnospiraceae bacterium oral taxon 082) [77]. At visit two where the mean CD4 count increased to 961 (± 572) fewer taxa were identified to be predictive of CD4 count but included some overlap between taxa identified in visit one including *L. wadei, Capnocytophaga* sp. oral taxon 412, Lachnospiraceae oral taxon 107 str. F0167, *C.* sp. FDAARGOS 737, and *Aggregatibacter* sp. 2125159857. Additionally, *T. phagedenis*, a non-pathogenic spirochete that is not considered to be a resident oral taxon, but is closely related to the periodontal pathogen *T. denticola* [79, 80], was found to be associated with low CD4 counts among children living with HIV in both visit one and visit two. While there are fewer pathogenic taxa contributing to the microbial signature of CD4 counts in visit two, *Selenomonas sputigena*, recently classified as a pathobiont capable of exacerbating the acidogenic activity of *S. mutans* in early childhood caries is highly weighted in the correlation coefficients driving lower CD4 counts [77]. Finally, while the mean CD4 count among children living with HIV drops at visit three (850 ± 521), the correlation coefficient between the relative balance of abundance between specific taxonomic groups is relatively weak (R = 0.38, p = 4.3e-09).

## DISCUSSION

Our results suggest that the temporal and spatial dynamics of the supragingival plaque microbiome are altered by HIV infection and exposure. We hypothesize that these alterations are a consequence of a combination of factors including: (1) reduced salivary flow and increased salivary pH among children in this cohort, (2) differences in taxonomic plasticity and cohesion of the plaque community over time, and (3) immune-mediated alterations to the oral community. Moreover, our longitudinal approach bolsters previous observations [28, 57] that while *S. mutans* is a dominant member of the supragingival plaque community during caries intensification, it is not involved or does not play a major role in the initiation of tooth decay. Instead, our results suggest that within-species strain-level functional diversity may be a catalyst for community dysbiosis and tooth decay inception. Further strain-level functional analyses are required to elucidate the role of the supragingival plaque community in the initiation of tooth decay.

### Reduced salivary flow and increased salivary pH are key contributors to alterations in the spatiotemporal structure of the supragingival plaque microbiome.

Whole saliva is a key modulator of the oral microbiome both physically and biochemically. On average, a healthy individual produces 0.6 liters of saliva per day which continuously coats the oral tissues and helps to clear debris, desquamated epithelial cells, and microorganisms [81, 82]. Saliva provides several key benefits essential for maintaining homeostasis of the oral cavity including lubrication to facilitate swallowing and removal of food particles from the oral tissues, digestive enzymes including amylase for the digestion of carbohydrates into sugars, and antimicrobial compounds including hydrogen peroxide, lactoferrin, lysozymes, and a variety of antimicrobial peptides that modulate the colonization of oral tissues by microbes [83–85]. Saliva also coats the teeth and other oral tissues with proteins and glycoproteins used by microbes to colonize the oral cavity, it transports nutrients and other metabolic substrates to members of the oral microbiome, and provides a moist environment with a stable pH (average ~ 6.7) that favors bacterial growth [82, 86].

People living with HIV often experience chronic dry mouth (xerostomia), and children especially are affected by HIV-associated salivary gland disease (HIV-SGD) [87]. HIV-SGD affects the parotid, submandibular, and sublingual salivary glands and results in decreased saliva production and poorer quality saliva including decreased levels of sodium, calcium chloride, cystatin (essential for tooth remineralization), and lysozymes [87, 88]. Comparisons of unstimulated salivary flow rates among children in this cohort found significant differences between HIV status groups. HUU children had the highest mean rate of salivary flow (0.33 ml/min) and HI children the lowest (0.22 ml/min) [37]. Importantly, the mean rate of salivary flow among HEU children was intermediate between HI and HUU at 0.27 ml/min [37], which may reflect long-lasting effects of the mother’s immune status on the oral biology of this growing cohort of children. Salivary gland hypofunction is associated with an increased risk of several oral diseases including caries disease and candidiasis [82, 89, 90]. Moreover, the mean pH of HI children in this cohort was significantly lower than HEU or HUU children which has important implications for the risk for tooth decay [37].

Given the biochemical properties of saliva and their importance in structuring the oral microbiome, we hypothesize that saliva is a primary contributor to differences in the biogeography of the supragingival plaque microbiome reported here. Location of individual teeth in the oral cavity, their proximity to major salivary ducts, morphological characteristics, and mechanical use (e.g., chewing vs tearing) influences their community composition. Previous research has found that the structure of the oral community follows an ecological gradient from the back to the front of the dentition so that the microbiome inhabiting the molars is distinct from that on the incisors [91–94]. This ecological pattern is clearly supported in the current study among HUU and HEU children. Conversely, individuals with impaired salivary flow due to injury or disease (e.g., Sjögren’s syndrome, see Proctor et al. [93]), experience a breakdown of this ecological gradient and a homogenization of the anterior and posterior oral communities, as is evident in our HI cohort. Interestingly, while the differences between the oral microbiome inhabiting anterior and posterior teeth in HEU children in the current study are significant, the distribution of these two communities is somewhat intermediate between HUU and HI children (see Fig. 4c), which may be reflective of their intermediate status in terms of salivary flow [37]. Previous studies have documented that disruptions in the natural ecological barriers that separate the human oral-pharyngeal and gut microbiome are symptomatic of disease [95, 96]. The results of this study suggest that these disruptions occur at much smaller spatial scales within the same host-associated habitat (i.e., the oral cavity) with possibly detrimental consequences towards overall oral and systemic health outcomes in HI children.

### Taxonomic turnover in the supragingival plaque microbiome may be adaptive response to changing host factors that is suppressed in disease.

Using distance-based taxonomic turnover analyses we found that the community inhabiting a single tooth may experience substantial taxonomic turnover over time, particularly among HUU children. This contrasts with expectations from previous longitudinal analyses of human-associated microbial ecosystems (primarily the gut microbiome) wherein large-scale shifts in the bacterial community is typically associated with illness or stress [49, 97, 98]. Given that bacteria inhabiting the oral cavity are subject to more direct and regular exchange with the external environment, it is possible that taxonomic turnover in the supragingival plaque microbiome is a signal of community health. Specifically, that temporal flexibility is reflective of the community’s ability to rapidly adapt to environmental perturbations. Previous investigations into the temporal stability of the oral microbiome have suggested that it is more dynamic than other human-associated microbial habitats as well as more influenced by the individual’s behavior and environment than purely genetic determinants of the composition of the oral microbiome [6, 8, 11, 12]. Other studies refute this, however, and argue that the oral microbiome is relatively stable over time [99, 100]. The results presented here strongly suggest that community fluctuations over time are dependent on both the health status of the individual as well as the scale in which these observations are made. Importantly, however, our results document a strong association between low taxonomic turnover and cariogenic taxa, including *S. mutans, P. multisaccharivorax*, and *P. denticola*, which may indicate that diseased oral communities experience less change over time due to a lack of starting diversity driven by acidogenic/aciduric taxa.

Despite substantial turnover on the individual tooth level, we do find clusters of taxa that are consistently co-associated over time within and across our HIV status groups. Thus, despite individualistic fluctuations in the overall community composition, a stable core of taxa is consistently present in the supragingival plaque microbiome, a pattern previously observed in longitudinal sampling of the oral cavity [8]. Clusters identified in the current study have similar functional expectations in that species that are associated with health or disease are consistently co-associated over time. For example, in our global core-association network, Cluster 6 is made exclusively of known or suspected cariogenic taxa while Cluster 1, Cluster 2, Cluster 5, and Cluster 4 are composed primarily of oral symbionts and structural taxa. Importantly, while modularity (a measure of community cohesion over time) is roughly equivalent among HI, HEU, and HUU children, there are notable differences in the structure and composition of core-association networks among these three groups. The most conspicuous of these differences is the lack of Cluster 6 and prominence of Cluster 2 among HEU children. Given that Cluster 6 is composed of cariogenic taxa, its absence in part may explain why, despite experiencing poor health outcomes in other areas that are similar to HI children [29–35], HEU children do not have a higher caries burden as compared to HUU children [25, 101, 102]. Importantly, however, Cluster 2, which is prominent in HEU children as compared to HI and HUU children, contains taxa typically associated with periodontal disease even though no children in this cohort exhibited symptoms of periodontal disease. It is unclear if these results suggest future periodontal disease risk among HEU children.

### Immune status among children living with HIV is associated with the abundance of cariogenic taxa.

Individual immune status among children living with HIV has previously been associated with the prevalence and severity of caries disease (see, for example [23]). In agreement with these findings, we find that low CD4 counts are strongly associated with the abundance of cariogenic taxa. Importantly, however, we also find that CD4 counts vary substantially among children living with HIV and that the mean CD4 count within this group fluctuates significantly over time. For example, we detect a significant increase in CD4 counts from visit one to visit two among children living with HIV (possibly the result of continued antiretroviral treatment), followed by a slight but significant decrease between visits two and three, a pattern also observed in HUU, but not HEU children. As CD4 counts generally decrease as children age [103], these patterns may be part of the normal aging process, however, given the impact of host immune status on the health of the oral cavity, we expect that continual monitoring of CD4 counts will be an important aspect of determining risk for tooth decay and other chronic health problems among HI children.

### Community dynamics before and after proliferation of S. mutans

Finally, we used random forest classification and post-hoc directionality analysis to better understand the community dynamics on a single tooth before and after high levels of *S. mutans* on the same tooth at different time periods. While our random forest classification model was unable to precisely classify the oral community before and after high levels of *S. mutans*, we did detect specific taxa that were present before high *S. mutans* that were not retained after the recolonization of the tooth post *S. mutans* colonization. In particular, non-mutans streptococcal species including *S. cristatus, S. sanguinis*, and *S. gordonii* were indicative of the community before but not after *S. mutans* colonization and proliferation. Importantly, however, these patterns are driven by a small subset of samples which may be indicative of functional differences among strains of these non-mutans streptococci. For example, previous work by our group has found substantial functional diversity among members of the streptococci, including *S. sanguinis* [104]. Moreover, while *S. sanguinis* is typically associated with oral health, high resolution amplicon sequencing of *S. sanguinis* and other putatively commensal streptococci species has documented that some are strongly associated with later stages of caries disease [105]. Given that *S. sanguinis* is highly predictive of later *S. mutans* abundance among some of our samples, it may be the case that specific strains of *S. sanguinis* play a role in the initialization of caries while others are protective against caries [78]. Finally, our results suggest that the community inhabiting a tooth will not recover to its previous state after *S. mutans* is the dominant taxon. Monitoring these processes at smaller time scales may elucidate which taxa are predictive of the community before and after high levels of cariogenic taxa including *S. mutans.*

## CONCLUSIONS

The results of this study highlight the importance of scale – both temporally and spatially – in understanding the impact of the bacterial supragingival plaque community in the development of caries in the context of HIV infection and exposure. We find that HIV infection and exposure has significant impact on the temporal and spatial structuring of the oral microbiome which may be the result of altered salivary flow and pH, individual host dynamics, and impaired immune status of individual children. Significantly, we find that healthy children within our cohort exhibit relatively high taxonomic turnover of the supragingival plaque microbiome while maintaining cohesive and consistent groups of taxa over time. Additionally, we find that low taxonomic turnover is associated with higher frequencies of cariogenic taxa including *S. mutans*. Moreover, we find that HIV infection homogenizes the oral microbiome across the anterior and posterior dentition, with altered salivary function likely a key factor. A further appreciation of the temporal and spatial dynamics of the oral microbiome in health and disease may be necessary to identify molecular mechanisms of oral microbiome dysbiosis and the initiation, progression, and ultimate consequences of tooth decay among children living with and exposed to HIV.

## Data Availability

The datasets generated and analyzed during the current study are available in the European Nucleotide Archive repository under accession number PRJEB76179. All preprocessing and analysis scripts are available at https://github.com/aemann01/long_oral_microbiome and are archived at Zenodo under the DOI: 10.5281/zenodo.11396312.

## Abbreviations

HUU: Child unexposed and therefore uninfected with HIV
HEU: Child exposed to but uninfected with HIV
HI: Child living with HIV
H-CF: A healthy tooth from a caries-free mouth
H-CE: A healthy tooth from a mouth with active enamel lesions
H-CD: Healthy tooth from a mouth with active dentin caries
E-CE: Tooth with an enamel lesion from a mouth with active enamel caries
E-CD: Tooth with an enamel lesion from a mouth with active dentin caries
D-CD: Tooth with a dentin cavity from a mouth with active dentin cavities
